# ﻿Karyotype and genome size variation in *Delphinium* subg. *Anthriscifolium* (Ranunculaceae)

**DOI:** 10.3897/phytokeys.234.108841

**Published:** 2023-10-18

**Authors:** Xiao-Yu Luo, Tang-Jie Nie, Heng Liu, Xue-Fei Ding, Ying Huang, Chun-Ce Guo, Wen-Gen Zhang

**Affiliations:** 1 Forestry College, Jiangxi Agricultural University, Nanchang 330045, China Jiangxi Agricultural University Nanchang China; 2 Jiangxi Provincial Key Laboratory for Bamboo Germplasm Resources and Utilization, Nanchang 330045, China Jiangxi Provincial Key Laboratory for Bamboo Germplasm Resources and Utilization Nanchang China; 3 Co-Innovation Center for Sustainable Forestry in Southern China, College of Biology and the Environment, Nanjing Forestry University, Nanjing 210037, China Nanjing Forestry University Nanjing China

**Keywords:** Columbines, *Consolida*, genome size, karyotype, ploidy, Ranunculales

## Abstract

Five taxa of Delphiniumsubg.Anthriscifolium have been karyologically studied through chromosome counting, chromosomal measurement, and karyotype symmetry. Each taxon that we investigated has a basic chromosome number of x = 8, D.anthriscifoliumvar.savatieri, D.anthriscifoliumvar.majus, *D.ecalcaratum*, and *D.callichromum* were diploid with 2n = 16, while D.anthriscifoliumvar.anthriscifolium was tetraploid with 2n = 32. Monoploid chromosome sets of the investigated diploid taxa contained 1 metacentric chromosome, 3 submetacentric chromosomes, and 4 subtelocentric chromosomes. Higher interchromosomal asymmetry (CV_CL_) was present in *D.ecalcaratum* and *D.callichromum* than in other taxa. The highest levels of intrachromosomal asymmetry (M_CA_) and heterogeneity in centromere position (CV_CI_) were found in D.anthriscifoliumvar.majus. Diploid and tetraploid genome sizes varied by 3.02–3.92 pg and 6.04–6.60 pg, respectively. Karyotype and genome size of D.anthriscifoliumvar.savatieri, D.anthriscifoliumvar.majus, *D.callichromum*, and *D.ecalcaratum* were reported for the first time. Finally, based on cytological and morphological data, the classification of *Delphiniumanthriscifolium* was revised.

## ﻿Introduction

*Delphinium* L., ca. 385 species and 232 species in China (Ilarslan et al. 1997; Wang 2019; Hadidchi et al. 2020), is a species-abundant genus of tribe Delphinieae in the buttercup family (Ranunculaceae) with great economic importance in terms of both horticultural and pharmaceutical value (Ghimire et al. 2015; Wang 2019; Wang et al. 2020). It is usually characterised by the following key traits: (1) In the zygomorphic flower, there are 5 petaloid sepals, with the upper one spurred; (2) a pair of dorsal petals are sessile, free, and spurred in the upper sepal, while a couple of lateral petals (i.e., staminodes) are spurless, each with a slender claw and an expanded limb; (3) follicles 3 (Tamura 1993; Wang and Warnock 2001; Wang 2019). Except for a few species found in tropical Africa’s montane regions, the genus is widely distributed in northern temperate regions (Milne-Redhead and Turrill 1952; Chartier et al. 2016; Aleem et al. 2020; Kashin et al. 2021).

To date, the classification of subgenus or groups in *Delphinium* is still controversial. For example, Wang (2019, 2020) divided DelphiniumintosubgenusDelphinastrum (DC.) Peterm. comprising sections *Aconitoides* W.T.Wang, *Elaopsis* Huth, *Delphinastrum* DC. and OligophyllonDimitrova, andsubgenusDelphiniumwithsectionAnthriscifolium. However, molecular phylogenetic studies indicated at least four monophyletic subgenera [i.e., D.subg.Consolida (DC.) Huth, subg. Delphinium, subg. Anthriscifolium (W.T.Wang) Wei Wang] should be accepted (Jabbour and Renner 2011, 2012; Wang et al. 2013; Xiang et al. 2017; DuPasquier et al. 2021). Interestingly, the taxon, including *D.anthriscifolium* Hance, is a monoclade, either a subgenus of *Delphinium* (Xiang et al. 2017) or an independent group included in *Delphinium* (Jabbour and Renner 2012; Wang et al. 2013).

As a recently erected subgenus, Delphiniumsubg.Anthriscifolium, including ca. 3 species [i.e., *D.anthriscifolium* Hance, *D.ecalcaratum* S.Y.Wang & K.F.Zhou, and *D.callichromum* Q.L.Gan & X.W.Li], is endemic to East Asia and mainly distributed in the south of Zhongtiao Mountain and Qinling Mountain in China (Ding et al. 1981; Gan and Li 2017; Wang 2019). Moreover, there are three varieties of *D.anthriscifolium* [i.e., D.anthriscifoliumvar.anthriscifolium, D.anthriscifoliumvar.majus Pamp., and D.anthriscifoliumvar.savatieri (Franch) Munz], among which there are obvious differences in flower size, colour, and shape, which cause disagreements in the taxonomic circumscription of this species and associated varieties.

Genome size refers to the amount of DNA contained in the gametes of a species, which is broadly constant within an organism and is primarily indicated by C-value (Pellicer et al. 2018; Twyman and Wisden 2018; Kocjan et al. 2022). C-value estimation is not only crucial for genomic sequencing and analysis (Gregory 2005) but also significant for the identification of species and taxonomic positions (Bourge et al. 2018; Sliwinska 2018). Furthermore, as an important character of genetic material, karyotype, including chromosome number, morphology, length, band type, and centromere position (de Resende 2017; Ning et al. 2018; Vimala et al. 2021; Mahmoudi and Mirzaghaderi 2023), was extensively used in the systematic and evolutionary study of plants (Baltisberger and Hörandl 2016; Peruzzi et al. 2017; Wang et al. 2020). So far, there are few reports on the genome size and karyotype of Delphiniumsubg.Anthriscifolium.

Here, we aim to: (1) determine the chromosome number, karyotype, and genome size of the above five taxa (i.e., D.anthriscifoliumvar.anthriscifolium, D.anthriscifoliumvar.majus, D.anthriscifoliumvar.savatieri, *D.ecalcaratum*, and *D.callichromum*); (2) evaluate the reliability of flow cytometry in genome size determination to infer ploidy levels in D.subg.Anthriscifolium; and (3) provide cytological evidence for the taxonomic revision of *D.anthriscifolium*.

## ﻿Materials and methods

### ﻿Sampling

**Figure 1.**
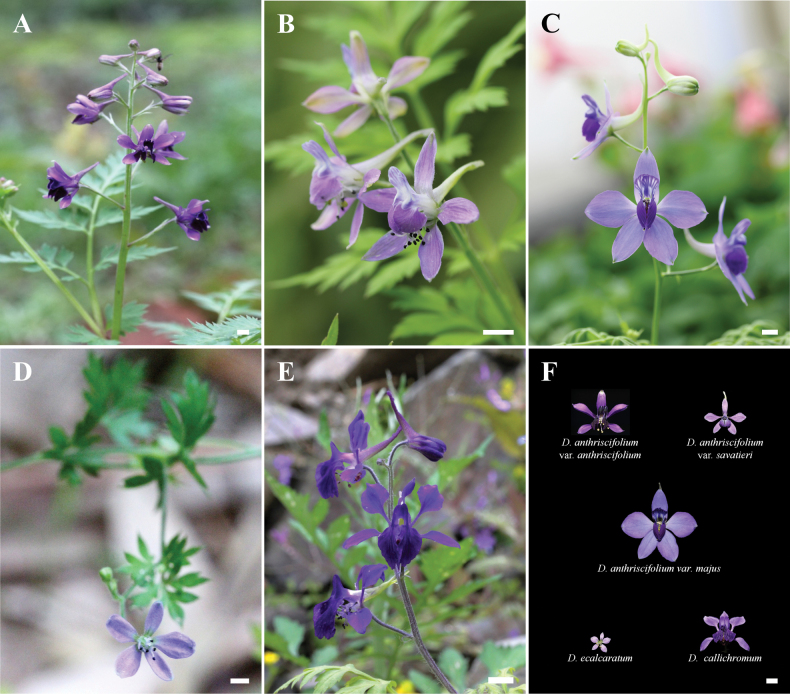
Five taxa of Delphiniumsubg.Anthriscifolium**A**D.anthriscifoliumvar.anthriscifolium**B**D.anthriscifoliumvar.savatieri**C**D.anthriscifoliumvar.majus**D***D.ecalcaratum***E***D.callichromum***F** flower front view of the above five taxa. Scale bars: 5 mm.

Materials of Delphiniumsubg.Anthriscifolium, including *D.ecalcaratum*, *D.callichromum*, *D.anthriscifolium* and its varieties (Fig. 1), were collected by field investigations in China during 2017–2021 (see Table 1 in detail), of which representatives were transplanted to the garden of Jiangxi Agricultural University. All vouchers were deposited in the herbarium of the College of Forestry, Jiangxi Agricultural University, China (**JXAU**).

**Table 1.** Chromosome number, ploidy, and genome size of Delphiniumsubg.Anthriscifolium in the study.

Pop	Taxa	Voucher information	2n	Ploidy	2C (pg)	1Cx (pg)
1	D.anthriscifoliumvar.anthriscifolium	Bamboo Culture Park, Yifeng County, Jiangxi, China, 28°24'31"N, 114°50'3"E, 24 Apr 2018, *Liu 1824*	32	4x	6.26	1.57
2	D.anthriscifoliumvar.anthriscifolium	Huacheng Temple, Yichun City, Jiangxi, China, 27°48'40"N, 114°22'44"E, 17 Apr 2019, *Zhang 1917*	32	4x	6.20	1.55
3	D.anthriscifoliumvar.anthriscifolium	Guling Town, Lushan City, Jiangxi, China, 29°34'28"N, 115°59'19"E, 17 Apr 2019, *Zhang 1904*	32	4x	6.33	1.58
4	D.anthriscifoliumvar.anthriscifolium	Miaofeng Mountain, Fuzhou City, Fujian, China, 26°4'53"N, 119°14'59"E, 2 May 2017, *Luo 1705*	32	4x	6.39	1.60
5	D.anthriscifoliumvar.anthriscifolium	Jiaoqiao Town, Nanchang City, Jiangxi, China, 28°46'6"N, 115°50'22"E, 16 Apr 2018, *Liu 1816*	32	4x*	6.13	1.53
6	D.anthriscifoliumvar.anthriscifolium	Fujia County, Fuzhou City, Jiangxi, China, 27°45'40"N, 116°26'17"E, 17 Apr 2019, *Nie 1917*	32	4x*	6.22	1.56
7	D.anthriscifoliumvar.anthriscifolium	Shangli County, Pingxiang City, Jiangxi, China, 27°50'37"N, 113°49'15"E, 17 Apr 2019, *Zhang 1918*	32	4x	6.04	1.51
8	D.anthriscifoliumvar.anthriscifolium	Guangxi Botanical Institute, Guangxi, China, 25°4'58"N, 110°18'45"E, 26 Mar 2020, *Zhang 2026*	32	4x	6.60	1.65
9	D.anthriscifoliumvar.savatieri	Hanfeng, Liuyang County, Shaanxi, China, 33°20'26"N, 105°59'43"E, 11 Apr 2020, *Gao 2011*	16	2x	3.32	1.66
10	D.anthriscifoliumvar.savatieri	Baisha River, Zhuxi County, Hubei, China, 32°5'27"N, 109°55'25"E, 18 Apr 2019, *Zhang 1818*	16	2x*	3.36	1.68
11	D.anthriscifoliumvar.savatieri	Sun Yat-sen Mausoleum, Nanjing City, Jiangsu, China, 32°5'23"N, 118°52'28"E, 19 Apr 2019, *Nie 1919*	16	2x	3.40	1.70
12	D.anthriscifoliumvar.savatieri	Baohua Mountain, Gourong City, Jiangsu, China, 32°8'8"N, 119°5'40"E, 19 Apr 2019, *Nie 1920*	16	2x*	3.43	1.72
13	D.anthriscifoliumvar.savatieri	Nanjing Zhongshan Botanical Garden, Jiangsu, China, 32°3'38"N, 118°50'16"E, 19 Apr 2019, *Nie 192*1	16	2x*	3.36	1.68
14	D.anthriscifoliumvar.savatieri	Zhongtiao Mountain, Yuncheng City, Shanxi, China, 32°46'44"N, 107°34'30"E, 21 May 2019, *Ren 1921*	16	2x	3.32	1.66
15	D.anthriscifoliumvar.savatieri	Jiaoqiao Town, Nanchang City, Jiangxi, China, 28°46'6"N, 115°50'22"E, 15 May 2021, *Luo 2115*	16	2x	3.31	1.66
16	D.anthriscifoliumvar.majus	Hefeng County, Enshi City, Hubei, China, 30°3'57"N, 110°8'45"E, 18 Apr 2019, *Zhang 1919*	16	2x	3.92	1.96
17	D.anthriscifoliumvar.majus	Songbai Town, Shennongjia, Hubei, China, 31°45'11"N, 110°40'5"E, 18 Apr 2019, *Zhang 192*5	16	2x*	3.80	1.90
18	D.anthriscifoliumvar.majus	Jiaoqiao Town, Nanchang City, Jiangxi, China, 28°46'6"N, 115°50'22"E, 15 May 2021, *Luo 2116*	16	2x	3.75	1.88
19	*D.ecalcaratum*	Jiaoqiao Town, Nanchang City, Jiangxi, China, 28°46'6"N, 115°50'22"E, 15 May 2021, *Luo 211*7	16	2x	3.02	1.51
20	*D.ecalcaratum*	Lingshan Mountain, Xinyang City, Henan, China, 31°54'46"N, 114°13'19"E, 19 Apr 2019, *Luo 191*9	16	2x*	3.03	1.52
21	*D.callichromum*	Baisha River, Zhuxi County, Hubei, China, 32°5'27"N, 109°55'25"E, 18 Apr 2019, *Luo 1918*	16	2x	3.10	1.55
22	*D.callichromum*	Jiaoqiao Town, Nanchang City, Jiangxi, China, 28°46'6"N, 115°50'22"E, 15 May 2021, *Luo 2118*	16	2x*	3.10	1.55

* Chromosome number and ploidy were validated by experimental analysis in the study, while others were inferred according to the genome size by flow cytometry. **Pop** = population.

### ﻿Flow cytometry (FCM) analysis

Twenty-two populations of Delphiniumsubg.Anthriscifolium were gathered with silica gel-dried leaves for the assessment of genome size by using flow cytometry (FCM; Table 1). In a petri dish containing pre-chilled MG^b^ dissociation solution, ca. 1 cm^2^ of leaf material was quickly chopped using a sharp blade. After 10 min on ice, the samples were filtered through a 40 μm filter into a tube with pre-chilled PI (50 μg/mL) and RNAase solution (50 μg/mL), which were then placed on ice and kept from light for 0.5 to 1 hour. Using BD FACSCalibur Flow Cytometer (USA), three replicates of each population of D.subg.Anthriscifolium were estimated with the internal standard (*Solanumlycopersicum* L., 900 M bp; The Tomato Genome Consortium 2012). According to Tian et al. (2011), the 2C-value of each sample was calculated as the fluorescence intensity ratio. To remove the effect of genome size resulting from recent polyploidisation, monoploid genome size value (1Cx; Greilhuber et al. 2005) was used and calculated through the 2C-value.

### ﻿Karyotype analysis

Somatic chromosomes were studied from the root tip cells of young seedlings. About 1–2 cm long roots were first pretreated in a 0.1% colchicine solution at 15 °C for 2–3 hours, then fixed in Carnoy I (absolute ethyl alcohol and glacial acetic acid in the proportions 3:1) for 30 minutes. After cleaning in purified water, they were hydrolysed in a mixture of 1 M HCl and 45% acetic acid (1:1) at 60 °C for 3–5 min and then stained with improved phenol magenta for 2 h. Five mitotic cells per species were examined and photographed using an Axio Imager A.1 microscope (Carl Zeiss, Germany) with ZEN software at 1000× magnification.

Short arm length (s) and long arm length (l) were measured using Image J (Collins 2007). Excel was used to determine additional chromosomal characteristics such as arm ratio (r = l/s), centromeric indices (CI), mean chromosome length (CL), relative chromosome length (RL), and total haploid length (THL). The coefficient of variation of chromosome length (CV_CL_) [(S_CL_ / X_CL_)× 100, where S_CL_: standard deviation; X_CL_: mean chromosome length] (Lavania and Srivastava 1992; Paszko 2006), coefficient of variation of the centromeric index (CV_CI_) [(S_CI_ / X_CI_) × 100, where S_CI_: standard deviation; X_CI_: mean centromeric index] (Paszko 2006), and mean centromeric asymmetry (M_CA_) (A × 100; the calculation of A is detailed in Watanabe et al. 1999) (Peruzzi and Eroğlu 2013) were calculated.

To infer the formulas of karyotype, the arm ratio (r), as defined by Levan et al. (1964), was used to categorise the chromosomes, and the homologous chromosome was allocated based on the similarity in length and centromere position using Photoshop CS6 software. The idiogram was constructed according to the arm ratio and relative length of the chromosomes. In order to illustrate karyotypic correlations between organisms, a bidimensional scatter plot was also created, in which the parameters CV_CL_ and M_CA_ are plotted on the x- and y-axes, respectively, and dots indicate each sample.

## ﻿Results

### ﻿Genome size of Delphiniumsubg.Anthriscifolium

**Figure 2.**
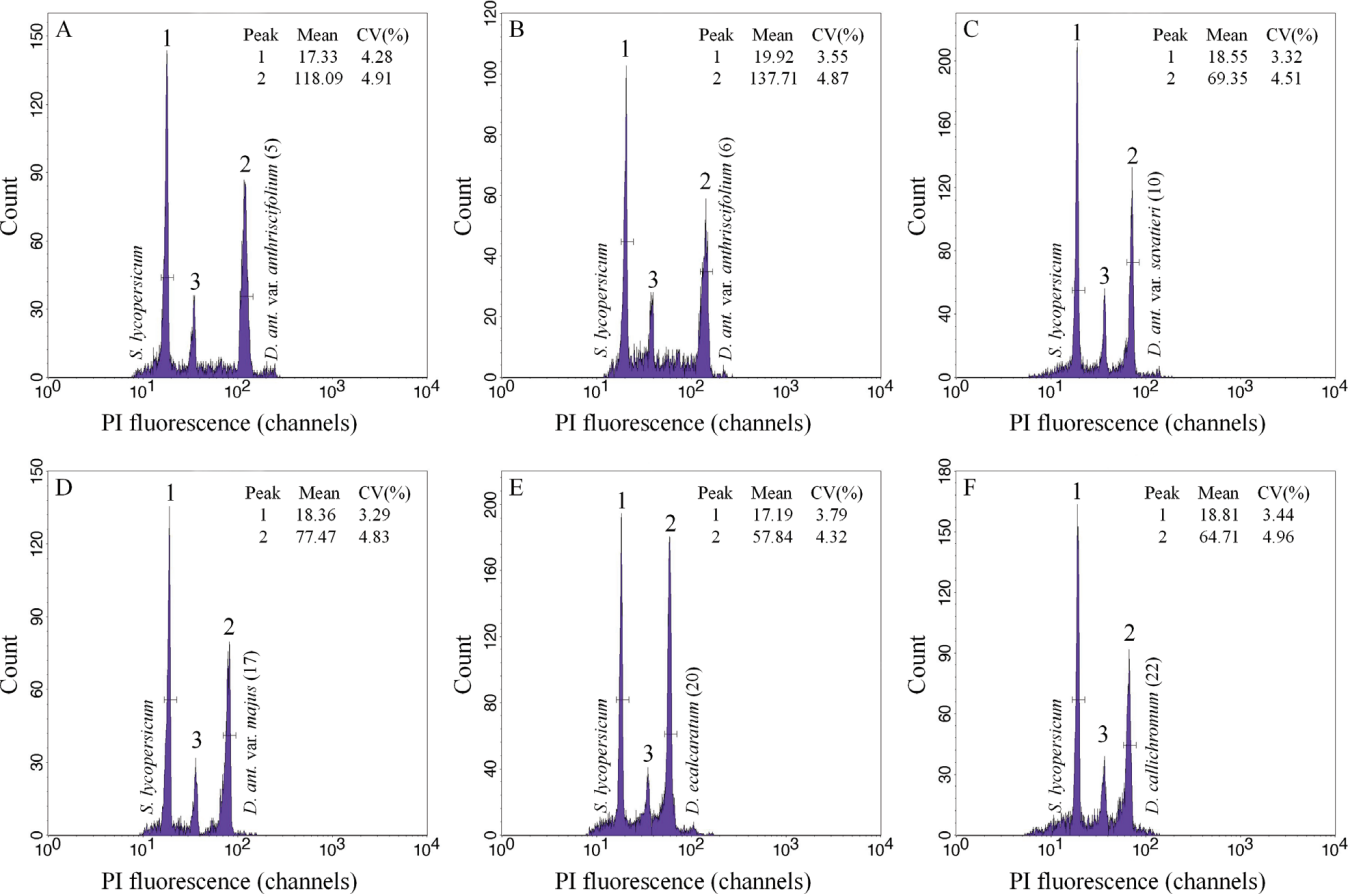
Flow cytometric histograms of Delphiniumsubg.Anthriscifolium was analysed simultaneously with the internal standard *Solanumlycopersicum*. In each histogram, the peaks are marked as follows: 1, nuclei of the internal standard at the G_1_ phase; 2, nuclei of the sample at the G_1_ phase. The mean channel number (PI fluorescence) and coefficient of variation value (CV, %) of each peak are also given; 3, nuclei of the internal standard at the G_2_ phase.

**Figure 3.**
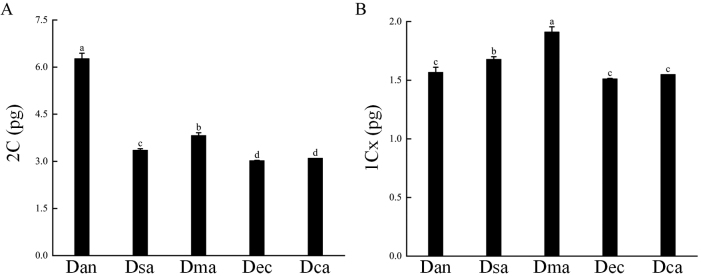
Comparison of the 2C and 1Cx mean values among Delphiniumsubg.Anthriscifolium. The columns marked with different index letters are significantly different at P < 0.05; those marked with the same index letters are not significantly different at P < 0.05 (one-way ANOVA followed by Tukey’s test). Error bars represent standard deviation.

In the FCM analysis, all studied taxa and the internal standards exhibited clear and sharp peaks (Fig. 2), and coefficients of variation were lower than 5%, supporting the reliability of the flow cytometric assessments. Twenty-two populations of D.subg.Anthriscifolium, including five taxa, i.e., D.anthriscifoliumvar.anthriscifolium, D.anthriscifoliumvar.savatieri, D.anthriscifoliumvar.majus, *D.ecalcaratum*, and *D.callichromum*, showed remarkable variation (3.02–6.60 pg) in genome size (Table 1). Nearly twice as large as the others, D.anthriscifoliumvar.anthriscifolium had the greatest 2C-values (6.27 ± 0.17 pg). In contrast, *D.ecalcaratum* (3.03 pg) and *D.callichromum* (3.10 pg) had the lowest values (Fig. 3A). The 1Cx values were highest in D.anthriscifoliumvar.majus (1.91 ± 0.04 pg), while lower in D.anthriscifoliumvar.anthriscifolium (1.57 ± 0.04 pg), *D.ecalcaratum* (1.51 pg), and *D.callichromum* (1.55 pg) (Fig. 3B). Additionally, the monoploid genome sizes of tetraploids (mean 1Cx = 1.57 pg) are smaller than those of diploids (mean 1Cx = 1.69 pg). Thus, genome loss or duplication events have occurred in the evolution of D.subg.Anthriscifolium.

### ﻿Karyotypes of Delphiniumsubg.Anthriscifolium

**Figure 4.**
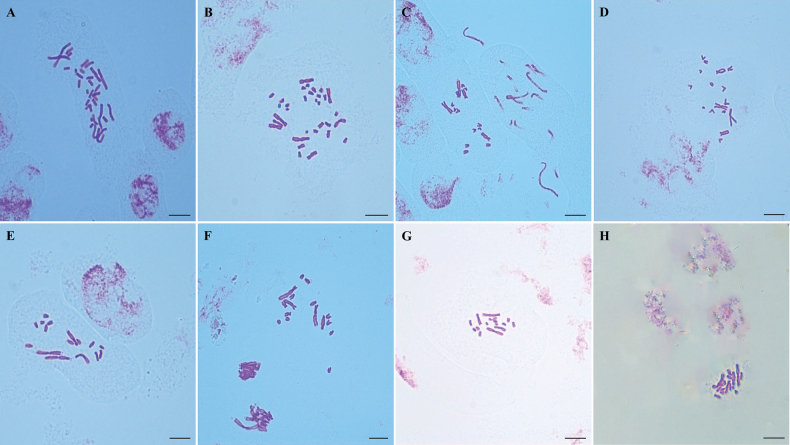
Somatic metaphases of Delphiniumsubg.Anthriscifolium**A**D.anthriscifoliumvar.anthriscifolium (5), 2n = 32 **B**D.anthriscifoliumvar.anthriscifolium (6), 2n = 32 **C**D.anthriscifoliumvar.savatieri (13), 2n = 16 **D**D.anthriscifoliumvar.savatieri (12), 2n = 16 **E**D.anthriscifoliumvar.savatieri (10), 2n = 16 **F**D.anthriscifoliumvar.majus (17), 2n = 16 **G***D.ecalcaratum* (20), 2n = 16 **H***D.callichromum* (22), 2n = 16. Numbers in brackets represented populations shown in Table 1. Scale bars: 10 μm.

**Figure 5.**
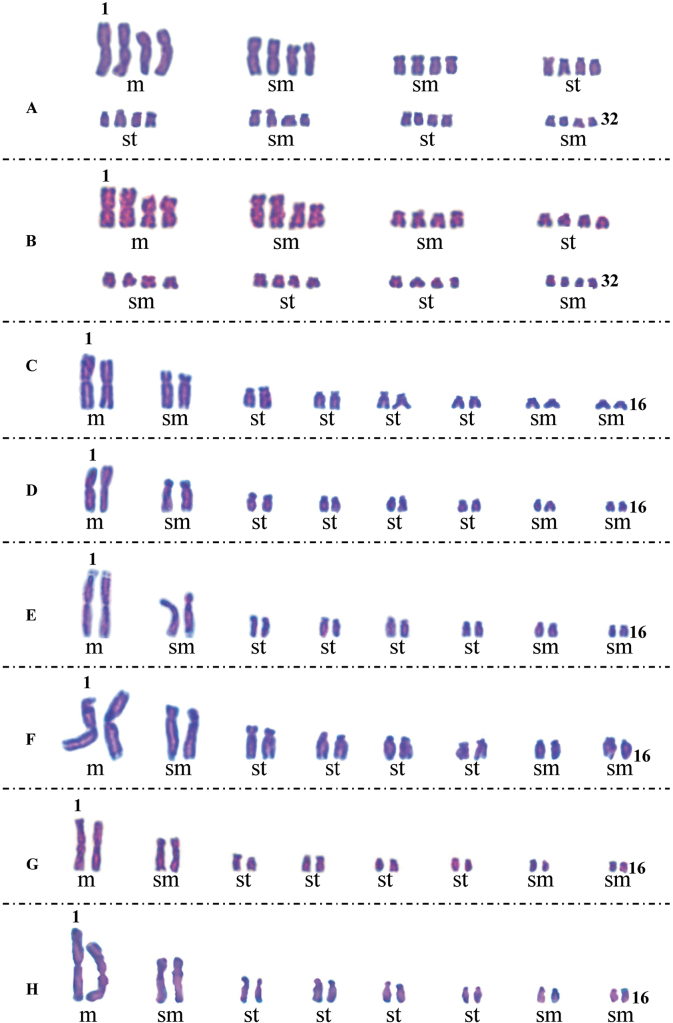
Karyotypes of Delphiniumsubg.Anthriscifolium**A**D.anthriscifoliumvar.anthriscifolium (5), 2n = 32 **B**D.anthriscifoliumvar.anthriscifolium (6), 2n = 32 **C**D.anthriscifoliumvar.savatieri (13), 2n = 16 **D**D.anthriscifoliumvar.savatieri (12), 2n = 16 **E**D.anthriscifoliumvar.savatieri (10), 2n = 16 **F**D.anthriscifoliumvar.majus (17), 2n = 16 **G***D.ecalcaratum* (20), 2n = 16 **H***D.callichromum* (22), 2n = 16. Numbers in brackets represented populations shown in Table 1. **m** = metacentric chromosome; **sat** = satellite chromosome; **sm** = submetacentric chromosome; **st** = subtelocentric chromosome.

**Figure 6.**
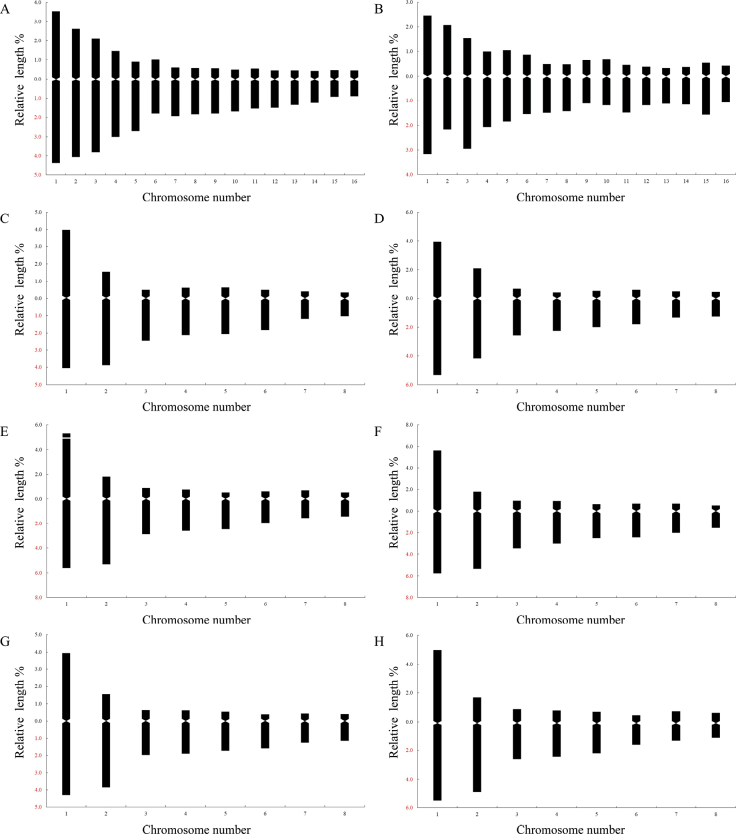
Haploid idiograms of Delphiniumsubg.Anthriscifolium**A**D.anthriscifoliumvar.anthriscifolium (5) **B**D.anthriscifoliumvar.anthriscifolium (6) **C**D.anthriscifoliumvar.savatieri (13) **D**D.anthriscifoliumvar.savatieri (12) **E**D.anthriscifoliumvar.savatieri (10) **F**D.anthriscifoliumvar.majus (17) **G***D.ecalcaratum* (20) **H***D.callichromum* (22). Numbers in brackets represented populations shown in Table 1.

Eight representative populations of D.subg.Anthriscifolium, including the above five taxa, were karyologically studied. Karyomorphometric data, microphotographs of metaphase plates, and idiograms are presented here (Tables 1–3; Figs 4–6).

#### ﻿1. Delphiniumanthriscifoliumvar.anthriscifolium

In two populations (Pop 5 and Pop 6) of D.anthriscifoliumvar.anthriscifolium from Jiangxi, China, the somatic and basic chromosome numbers were 2n = 32 and x = 8, respectively (Table 1; Fig. 4A, B). Two pairs of chromosomes (i.e., I–II) are metacentric, eight pairs (i.e., III–VI, XI–XII, and XV–XVI) are submetacentric, and six pairs (i.e., VII–X and XIII–XIV) are subtelocentric (Tables 2, 3; Figs 5A, B, 6A, B). Thus, the karyotype formula is 2n = 4x = 32 = 4m + 16sm + 12st.

#### ﻿2. Delphiniumanthriscifoliumvar.savatieri

In three populations (i.e., Pop 10 from Hubei, Pop 12 and Pop 13 from Jiangsu), the somatic and basic chromosome numbers are 2n = 16 and x = 8, respectively (Table 1; Fig. 4C–E). Pop 12 and Pop 13 have more similar karyotypes: one pair of chromosomes (i.e., I) is metacentric, three pairs (i.e., II, VII, and VIII) are submetacentric, and four pairs (i.e., III–VI) are subtelocentric (Tables 2 and 3; Figs 5C, D, 6C, D). The karyotype formula is 2n = 2x = 16 = 2m + 6sm + 8st. However, Pop 10 differed from Pop 12 and Pop 13 in that it has a secondary constriction on the first pair of chromosomes (Figs 5E, 6E), so its karyotype formula is 2n = 2x = 16 = 2m^sat^ + 6sm + 8st.

**Table 2.** Karyomorphological parameters of Delphiniumsubg.Anthriscifolium in the study.

Taxa	Pop	Chromosome pair	CL (µm)	r	CI	RL (%)	Type
D.anthriscifoliumvar.anthriscifolium	5	I	7.42 ± 0.72	1.28 ± 0.06	0.44	14.47	m
		II	6.35 ± 0.48	1.59 ± 0.05	0.39	12.38	m
		III	5.85 ± 0.12	2.10 ± 0.42	0.33	11.40	sm
		IV	4.28 ± 0.25	2.45 ± 0.55	0.29	8.35	sm
		V	3.25 ± 0.50	2.79 ± 0.26	0.26	6.34	sm
		VI	2.76 ± 0.08	2.06 ± 0.42	0.33	5.38	sm
		VII	2.54 ± 0.02	3.34 ± 0.21	0.23	4.95	st
		VIII	2.41 ± 0.02	3.15 ± 0.00	0.24	4.71	st
		IX	2.34 ± 0.03	3.38 ± 0.27	0.23	4.57	st
		X	2.19 ± 0.01	3.26 ± 0.13	0.23	4.27	st
		XI	2.06 ± 0.07	2.88 ± 0.19	0.26	4.01	sm
		XII	1.95 ± 0.02	2.73 ± 0.76	0.20	3.79	sm
		XIII	1.76 ± 0.08	3.02 ± 0.06	0.25	3.42	st
		XIV	1.61 ± 0.09	3.08 ± 0.29	0.25	3.14	st
		XV	1.40 ± 0.01	1.82 ± 0.14	0.30	2.72	sm
		XVI	1.31 ± 0.06	1.88 ± 0.13	0.35	2.56	sm
	6	I	5.60 ± 0.05	1.22 ± 0.09	0.45	13.90	m
		II	4.14 ± 0.13	1.14 ± 0.13	0.47	10.27	m
		III	4.62 ± 0.16	1.90 ± 0.02	0.35	11.45	sm
		IV	3.43 ± 0.52	2.12 ± 0.03	0.32	8.50	sm
		V	2.75 ± 0.20	1.90 ± 0.17	0.35	6.81	sm
		VI	2.24 ± 0.23	2.33 ± 0.75	0.31	5.55	sm
		VII	1.81 ± 0.08	3.09 ± 0.04	0.33	4.49	st
		VIII	1.90 ± 0.04	3.01 ± 0.00	0.37	4.70	st
		IX	1.78 ± 0.29	2.03 ± 0.43	0.24	4.42	sm
		X	1.88 ± 0.04	1.73 ± 0.01	0.25	4.66	sm
		XI	1.76 ± 0.25	3.25 ± 0.01	0.24	4.37	st
		XII	1.56 ± 0.01	3.08 ± 0.07	0.24	3.86	st
		XIII	1.76 ± 0.50	3.23 ± 0.24	0.26	4.37	st
		XIV	1.32 ± 0.20	3.10 ± 0.02	0.28	3.28	st
		XV	1.59 ± 0.20	2.91 ± 0.06	0.24	3.94	sm
		XVI	1.68 ± 0.22	2.60 ± 0.12	0.24	4.16	sm
D.anthriscifoliumvar.savatieri	13	I	7.65 ± 0.53	1.05 ± 0.04	0.49	28.15	m
		II	5.14 ± 0.43	2.71 ± 0.31	0.27	18.91	sm
		III	2.87 ± 0.11	4.23 ± 0.91	0.19	10.54	st
		IV	2.73 ± 0.03	3.36 ± 0.03	0.23	10.05	st
		V	2.67 ± 0.03	3.60 ± 0.49	0.22	9.84	st
		VI	2.15 ± 0.26	3.53 ± 0.14	0.22	7.90	st
		VII	1.53 ± 0.08	2.90 ± 0.01	0.26	5.64	sm
		VIII	1.39 ± 0.00	2.90 ± 0.13	0.26	5.12	sm
	12	I	8.15 ± 1.58	1.24 ± 0.17	0.45	27.24	m
		II	6.14 ± 0.20	2.04 ± 0.06	0.33	20.52	sm
		III	3.13 ± 0.16	3.80 ± 0.01	0.21	10.45	st
		IV	2.62 ± 0.08	4.47 ± 1.41	0.19	8.75	st
		V	2.46 ± 0.09	3.75 ± 0.02	0.21	8.23	st
		VI	2.37 ± 0.02	3.05 ± 0.02	0.25	7.93	st
		VII	1.82 ± 0.00	2.82 ± 0.18	0.26	6.09	sm
		VIII	1.55 ± 0.24	2.89 ± 0.14	0.26	5.20	sm
D.anthriscifoliumvar.savatieri	10	I	10.43 ± 0.11	1.13 ± 0.03	0.47	30.34	m^sat^
		II	6.77 ± 0.49	2.95 ± 0.01	0.25	19.70	sm
		III	3.60 ± 0.16	4.31 ± 1.43	0.20	10.49	st
		IV	3.30 ± 0.03	3.25 ± 0.32	0.24	9.59	st
		V	2.89 ± 0.14	4.60 ± 0.23	0.18	8.40	st
		VI	2.45 ± 0.10	3.23 ± 0.02	0.24	7.14	st
		VII	2.18 ± 0.10	2.55 ± 0.40	0.28	6.36	sm
		VIII	1.84 ± 0.15	2.64 ± 0.18	0.28	5.34	sm
D.anthriscifoliumvar.majus	17	I	11.08 ± 0.48	1.05 ± 0.05	0.49	28.96	m
		II	7.10 ± 0.04	2.97 ± 0.01	0.25	18.58	sm
		III	4.40 ± 0.06	3.43 ± 0.10	0.23	11.51	st
		IV	3.75 ± 0.31	3.43 ± 0.34	0.23	9.81	st
		V	3.26 ± 0.15	4.75 ± 1.40	0.18	8.53	st
		VI	3.07 ± 0.00	4.03 ± 0.84	0.20	8.03	st
		VII	2.67 ± 0.05	2.86 ± 0.01	0.26	6.97	sm
		VIII	1.86 ± 0.31	2.84 ± 0.06	0.26	4.87	sm
*D.ecalcaratum*	20	I	8.17 ± 0.10	1.06 ± 0.05	0.49	30.99	m
		II	5.39 ± 0.03	2.66 ± 0.26	0.27	20.45	sm
		III	2.61 ± 0.02	3.14 ± 0.04	0.24	9.92	st
		IV	2.51 ± 0.04	3.12 ± 0.10	0.24	9.53	st
		V	2.27 ± 0.02	3.21 ± 0.09	0.24	8.62	st
		VI	1.95 ± 0.07	3.65 ± 0.72	0.22	7.38	st
		VII	1.70 ± 0.00	2.65 ± 0.31	0.28	6.43	sm
		VIII	1.50 ± 0.09	2.73 ± 0.09	0.27	5.70	sm
*D.callichromum*	22	I	9.47 ± 1.40	1.20 ± 0.14	0.46	29.21	m
		II	6.48 ± 0.12	2.50 ± 0.62	0.29	19.99	sm
		III	3.36 ± 0.17	3.25 ± 0.33	0.24	10.36	st
		IV	3.14 ± 0.10	3.45 ± 0.46	0.23	9.70	st
		V	2.56 ± 0.45	3.23 ± 0.07	0.24	7.90	st
		VI	2.07 ± 0.01	3.74 ± 0.25	0.21	6.38	st
		VII	1.96 ± 0.09	2.01 ± 0.27	0.33	6.05	sm
		VIII	1.66 ± 0.09	1.77 ± 0.01	0.36	5.11	sm

**CI** = centromeric index; **CL** = chromosome length, mean value ± standard deviation; **m** = metacentric chromosome; **Pop** = population, numbers shown in Table 1; **r** = arm ratio, mean value ± standard deviation; **RL** = relative chromosome length; **sat** = chromosome showing secondary constriction; **sm** = submetacentric chromosome; **st** = subtelocentric chromosome.

**Table 3.** Karyotype parameters of Delphiniumsubg.Anthriscifolium in the study.

Taxa	Pop	Ploidy	2n	Karyotype formula	THL	CV_CL_	M_CA_	CV_CI_
D.anthriscifoliumvar.anthriscifolium	5	4x	32	2n = 4m + 16sm + 12st	51.30	60.13	40.32	22.07
	6	4x	32	2n = 4m + 16sm + 12st	40.33	50.93	37.18	27.08
D.anthriscifoliumvar.savatieri	13	2x	16	2n = 2m + 6sm + 8st	27.18	62.81	46.58	34.01
	12	2x	16	2n = 2m + 6sm + 8st	29.91	65.20	47.18	30.83
	10	2x	16	2n = 2m^sat^+ 6sm + 8st	34.37	68.27	46.05	33.27
D.anthriscifoliumvar.majus	17	2x	16	2n = 2m + 6sm + 8st	38.24	63.10	47.59	35.79
*D.ecalcaratum*	20	2x	16	2n = 2m + 6sm + 8st	26.35	68.87	44.65	29.84
*D.callichromum*	22	2x	16	2n = 2m + 6sm + 8st	32.42	69.63	40.11	29.09

**CV_CI_** = Coefficient of Variation of Centromeric Index; **CV_CL_** = Coefficient of Variation of Chromosome Length; **m** = metacentric chromosome; **M_CA_** = Mean Centromeric Asymmetry; **Pop** = population, numbers shown in Table 1; **sat** = satellite chromosome; **sm** = submetacentric chromosome; **st** = subtelocentric chromosome; **THL** = total haploid length, µm.

#### ﻿3. Delphiniumanthriscifoliumvar.majus

In Pop 17, the somatic and basic chromosome numbers are 2n = 16 and x = 8, respectively (Table 1; Fig. 4F). Its chromosome set includes one pair of metacentric chromosomes (i.e., I), three submetacentric (i.e., II, VII, and VIII), and four subtelocentric chromosomes (i.e., III–VI; Tables 2, 3; Figs 5F, 6F). Hence, the karyotype formula is 2n = 2x = 16 = 2m + 6sm + 8st.

#### ﻿4. *Delphiniumecalcaratum*

In Pop 20 from Xinyang City of Henan, China, the somatic and basic chromosome numbers are 2n = 16 and x = 8, respectively (Table 1; Fig. 4G). One pair of metacentric chromosomes (i.e., I), three pairs of submetacentric chromosomes (i.e., II, VII, and VIII), and four subtelocentric chromosomes (i.e., III–VI) make up the chromosome set of *D.ecalcaratum* (Tables 2, 3; Figs 5G, 6G). Therefore, the karyotype formula is 2n = 2x = 16 = 2m + 6sm + 8st.

#### ﻿5. *Delphiniumcallichromum*

In Pop 22 collected from the type locality of Zhuxi County, Hubei, China, the somatic and basic chromosome numbers are 2n = 16 and x = 8, respectively (Table 1; Fig. 4H). Its chromosome set includes one pair of metacentric chromosomes (i.e., I), three submetacentric (i.e., II, VII, and VIII), and four subtelocentric chromosomes (i.e., III–VI) (Tables 2, 3; Figs 5H, 6H). Accordingly, the karyotype formula is 2n = 2x = 16 = 2m + 6sm + 8st.

### ﻿Karyotype asymmetry analysis

**Figure 7.**
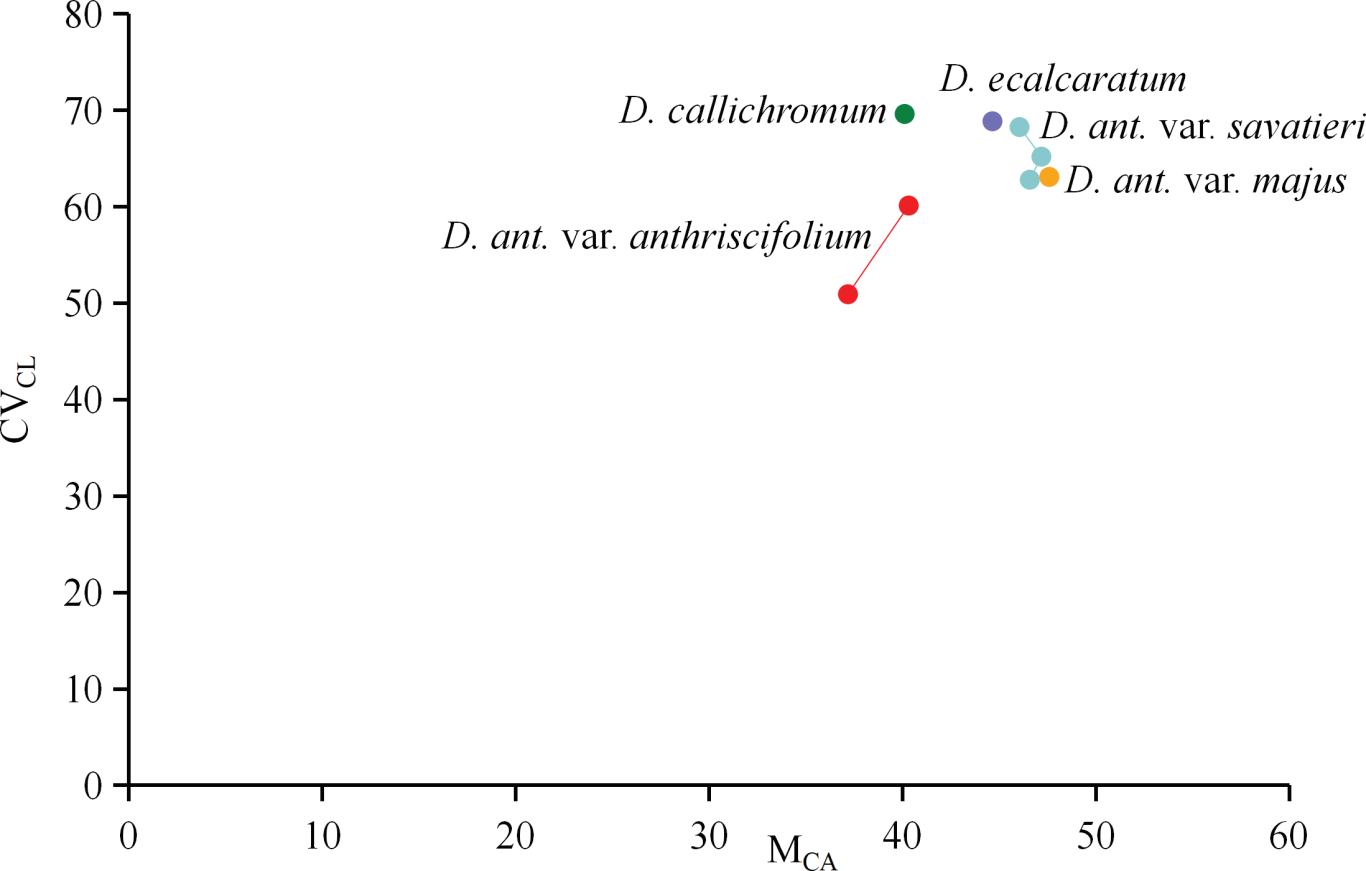
Scatter diagram of Delphiniumsubg.Anthriscifolium based on karyotype parameters **CV_CL_ vs. M_CA_. CV_CL_** = Coefficient of Variation of Chromosome Length; **M_CA_** = Mean Centromeric Asymmetry.

In all five taxa of Delphiniumsubg.Anthriscifolium, the total haploid length (THL) of *D.ecalcaratum* was probably the shortest (26.35), while that of D.anthriscifoliumvar.majus was the longest (up to 38.24). The highest level of interchromosomal asymmetry, estimated via CV_CL_, was found in *D.callichromum* (69.63). In contrast, the lowest level of CV_CL_ was found in D.anthriscifoliumvar.anthriscifolium (its mean value was 55.53). The highest values of both the heterogeneity in centromere position (CV_CI_) and intrachromosomal asymmetry (M_CA_) were found in D.anthriscifoliumvar.majus (47.59 and 35.79, respectively; Table 3). As seen in the scatter diagram (Fig. 7) drawn based on the parameter CV_CL_ vs M_CA_, compared to D.anthriscifoliumvar.anthriscifolium and *D.callichromum*, D.anthriscifoliumvar.savatieri, D.anthriscifoliumvar.majus, and *D.ecalcaratum* gathered together, indicating that they might be more closely related.

## ﻿Discussion

In Ranunculaceae, taxonomic position and evolutionary history were generally inferred by using chromosomal data (Tamura 1993; Yang 2001; Cires et al. 2010; Soza et al. 2013; Orooji et al. 2022). So far, nearly 60 species of *Delphinium* have been karyologically studied (Tjebbes 1927; Kolar et al. 2012; Gupta et al. 2018; Bosch et al. 2019; see www.iaptglobal.org/chromosome-data). The basic number of haploid chromosomes in *Delphinium* was typically 8 (Legro 1961; Orellana et al. 2007; Yuan and Yang 2008), with 9 (Blanché and Molero 1983; Bosch 1999; Bosch et al. 2002) and 10 (Sarkar et al. 1982) occasionally occurring in some circumstances. The chromosome number of most *Delphinium* plants was 2n = 16, while a few were 2n = 32, such as *D.denudatum* (Al-Kelidar and Richards 1981), *D.chrysotrichum* (Yuan 2006), and *D.spirocentrum* (Yuan and Yang 2008). Here, chromosome numbers of five taxa in D.subg.Anthriscifolium (i.e., D.anthriscifoliumvar.anthriscifolium, D.anthriscifoliumvar.majus, D.anthriscifoliumvar.savatieri, *D.ecalcaratum*, and *D.callichromum*) are reported. All studied taxa have a basic chromosome number of x = 8, D.anthriscifoliumvar.savatieri, D.anthriscifoliumvar.majus, *D.ecalcaratum*, and *D.callichromum* are diploid with 2n = 16, while D.anthriscifoliumvar.anthriscifolium is tetraploid with 2n = 32.

Furthermore, the karyotypes of *Delphinium* taxa were very consistent, typically consisting of one pair of large metacentric, one pair of large submetacentric, five pairs of medium-sized subtelocentric, and one pair of smaller submetacentric (rarely subtelocentric) chromosomes (Lewis et al. 1951; Yang 2001; Yuan and Yang 2008; Kolar et al. 2012). In the study, we found that the karyotype of the diploid cytotype in D.subg.Anthriscifolium shared the traits listed below: (1) the first pair (metacentric chromosomes) and the second pair (submetacentric chromosomes) of chromosomes are significantly larger than the remaining six pairs; (2) the proportion of subtelocentric chromosomes is relatively high; and (3) intrachromosomal asymmetry and interchromosomal asymmetry are both high. Two pairs of large metacentric, eight pairs of submetacentric, and six pairs of subtelocentric chromosomes make up the tetraploid cytotype in D.anthriscifoliumvar.anthriscifolium. The karyotype formula of D.anthriscifoliumvar.anthriscifolium is 2n = 4m + 16sm + 12st, consistent with the results of Yuan and Yang (2008). The karyotype formulas of D.anthriscifoliumvar.savatieri, D.anthriscifoliumvar.majus, *D.ecalcaratum*, and *D.callichromum* are 2n = 2m + 6sm + 8st, consistent with the karyotype formulas of *D.caeruleum*, *D.maximowiczii*, D.kamaoensevar.glabrescens, *D.nangchienense*, and D.candelabrumvar.monanthum (Yang 1996; Liu and Ho 1999).

On the genome size of Ranunculaceae, few related studies involving ten genera (i.e., *Ranunculus*, *Eranthis*, *Helleborus*, *Hepatica*, *Thalictrum*, *Delphinium*, *Anemone*, *Ficaria*, *Adonis*, and *Trollius*), showed that the 2C-value of diploid taxa significantly ranged from 0.5 to 57.3 pg and from 14.8 to 89.2 pg for tetraploid taxa (Zonneveld 2001; Mabuchi et al. 2005; Cires et al. 2009; Cires et al. 2010; Zonneveld 2010; Soza et al. 2013; Zonneveld 2015; Mitrenina et al. 2020; Mitrenina et al. 2021; Salvado et al. 2022; Seidl et al. 2022). According to Salvado et al.’s (2022) report on the genome size of *Delphinium*, the tetraploid *D.montanum* had a 1C value of 10.32 pg. Here, the 2C-value of D.subg.Anthriscifolium was 3.02–3.92 pg for diploids and 6.04–6.60 pg for tetraploids, respectively. Chromosome counts were completed for selected taxa to confirm ploidy and further calibrate the flow cytometry results. However, the above data lacks comparability due to the difference in experimental conditions and reference genome species.

Interestingly, in the study, the monoploid genome sizes of tetraploids (mean 1Cx = 1.57 pg) are less than those of diploids (mean 1Cx = 1.69 pg; see Fig. 3B), maybe showing a general tendency toward genome downsizing in the evolution of Delphiniumsubg.Anthriscifolium. Following polyploidisation, chromosome counts and genome size may change independently or dependently due to sequence loss and gain, chromosomal elimination, or chromosome fusions and fissions (Heslop-Harrison et al. 2023). Typically, the loss of repetitive DNA, such as retroelements or retrotransposons, caused the decline in monoploid genomes (Leitch and Bennett 2004; Bennetzen et al. 2005; Simonin and Roddy 2018). In addition, genome size data can be used to estimate ploidy in closely related taxa when properly calibrated with known cytological standards (Shearer and Ranney 2013; Lattier et al. 2014; Hembree et al. 2019). Delphiniumanthriscifoliumvar.anthriscifolium is tetraploid with a genome size of about 6.28 pg. In comparison, the remaining diploid taxa have a genome size of approximately 3.38 pg, meaning that polyploidisation occurred in the D.subg.Anthriscifolium.

### ﻿Taxonomic treatment

#### ﻿1. Delphinium anthriscifolium

Taxon classificationPlantaeRanunculales Ranunculaceae

Hance. In J. Bot. 5: 207. 1868.

716559F8-2FFF-564E-B792-251DD7A37673

= D.calleryi Franch. in Bull. Mens. De la Soc. Linn. De Paris, 1: 329. 1882. ≡ D.anthriscifoliumvar.calleryi (Franch.) Fin. & Gagnep. in Bull. Soc. Bot. Fr. 51: 471. 1904. syn. nov. Type: China: Aomen (Macao), 1841, *Callery 6* (Holotype P!); Aomen, 1844, *Callery 51* (Isotypes P!).
= D.cavaleriense Lévl. et Vant. in Bull. Acad. Géog. Bot. 11: 49. 1902., syn. nov. Type: China: Guizhou (Kweichow), “environs de Tou-chan, belles fleurs bleues”, 2 June 1898, *J. Cavalerie 2344* (Holotype E!; Isotypes K!).
= D.cerefolium Lévl. et Vant. in Bull. Acad. Géog. Bot. 11: 49. 1902., syn. nov. Type: China: Guizhou (Kouy-Tcheou), Guiyang (Kouy-Yang), “mont du College”, 2 June 1898, *Chaffanjon s.n.* (Holotype E!).

##### Type material.

***Lecotype***: China: Guangdong (Kwantung), “necnon prope rupem calcaream kai-kun-shek, secus eundem fluvium”, June 1867, *Sampson, Hance no. 10125* (Holotype K!; Isotypes BM! NY! P! JE! GH).

#### ﻿2. Delphinium savatieri

Taxon classificationPlantaeRanunculales Ranunculaceae

Franch. In Bull. Mens. De la Soc. Linn. De Paris 1: 330. 1882.

EF7AAEC6-7A1B-5FF2-8DE6-0DAD46FA781B

≡ D.anthriscifoliumvar.savatieri (Franchet) Munz., J. Armold Arbor. 48: 261. 1967. Type: China: Zhejiang (Tche-kiang/Chekiang), “in siccis ad pedem montium Shao-Shin, prope Ning-po”, May 1863, *Lud. Savatier* (Holotype P!; Isotype P!).
= D.robertianum Lévl. et Vant. in Bull. Acad. Géog. Bot. 11: 49. 1902., syn. nov. Type: China: Guizhou (Kouy-tcheou), Guiyang (Kouy-yang), 9 Dec 1897, *no. 2025* (Holotype E!).
= D.minutum Lévl. et Vant. in Bull. Herb. Boiss. sér. 2, 6: 505. 1906., syn. nov. Type: China: Guizhou, 2 Mar 1904, *Jos. Esquirol no. 23* (Holotype E!).
= D.kweichowense W.T.Wang in Acta Bot. Sin., 10: 283. 1962., syn. nov. Type: China: Guizhou, Huishui, 18 July 1930, *Y. Jiang 8571* (PE!).

##### Note.

Morphologically, *D.savatieri* differs from *D.anthriscifolium* in that the staminode limb is ovate (vs. dolabriform), 2-lobed (vs. 2-parted), and its base is broadly cuneate (vs. subtruncate). Cytologically, *D.savatieri* also differs from *D.anthriscifolium* in that its karyotype formula is 2n = 2x = 16 = 2m + 6sm + 8st (vs. 2n = 4x = 32 = 4m + 16sm + 12st).

#### ﻿3. Delphinium zanlanscianense

Taxon classificationPlantaeRanunculales Ranunculaceae

W.G.Zhang & X.Y.Luo nom. nov.

206A1250-92B9-5DD2-BA68-6CAA0D945E11

urn:lsid:ipni.org:names:77328947-1

≡ Delphiniumanthriscifoliumvar.majus Pamp. in Nuovo Giorn. Bot. Ital., n.s., 20: 288. 1915.
= D.anthriscifoliumf.latilobulatum W.T.Wang in Acta Bot. Sin., 10: 279. 1962., syn. nov. Type: China: Hunan, Xue-Feng-Shan, 1954, *Z. T. Li 2371* (Holotype PE!; Isotype PE!).

##### Type material.

***Lecotype***: China: Hubei (Hu-peh), Zhanglang County (Zan-lan-scian), 1913, *P. C. Silvestri no. 3917* (Holotype FI!).

##### Note.

Morphologically, D.anthriscifoliumvar.majus differs from D.anthriscifoliumvar.anthriscifolium in that the flowers are 2.3–3.4 cm long (vs. 1.0–1.8 cm), spur 1.7–2.2 cm (vs. 0.5–2.2 cm) and its base 3.0–4.0 mm (vs. 1.5–4.0 mm) in diam., other sepals 1.2–1.6 cm (vs. 0.6–1.6 cm), staminode limb broadly ovate (vs. dolabriform or ovate). Cytologically, D.anthriscifoliumvar.majus differs from D.anthriscifoliumvar.anthriscifolium in that its karyotype formula is 2n = 2x = 16 = 2m + 6sm + 8st (vs. 2n = 4x = 32 = 4m + 16sm + 12st).

When elevating D.anthriscifoliumvar.majus to the rank of species, the name is already occupied by *D.majus* (W.T.Wang) W.T.Wang (Wang and Hsiao 1965), making it necessary to propose a replacement name. Thus, we propose the name ‘*zanlanscianense*’ based on the locality of its lectotype.

## ﻿Conclusions

In the present study, comparative karyomorphological analyses and genome size determinations of five taxa of Delphiniumsubg.Anthriscifolium have been carried out. The chromosome numbers of *D.savatieri*, *D.zanlanscianense*, *D.callichromum*, and *D.ecalcaratum* were determined for the first time. Karyotypes of D.subg.Anthriscifolium were shown to have both common and species-specific features related to chromosome number, size, and morphology. All studied taxa have the basic chromosome numbers x = 8, diploid, or polyploid cytotypes, and the monoploid genome size (C-value) determined by flow cytometry varies more than twice. Additionally, the monoploid genome sizes of tetraploids (mean 1Cx = 1.57 pg) are smaller than those of diploids (mean 1Cx = 1.69 pg). Thus, genome loss or duplication events have occurred in the evolution of D.subg.Anthriscifolium. Finally, based on cytological and morphological evidence, D.anthriscifoliumvar.savatieri was restored to species rank, and D.anthriscifoliumvar.majus was elevated and renamed as *D.zanlanscianense*.

## Supplementary Material

XML Treatment for Delphinium anthriscifolium

XML Treatment for Delphinium savatieri

XML Treatment for Delphinium zanlanscianense

## ﻿Additional information

### Author ORCIDs

Xiao-Yu Luo https://orcid.org/0009-0005-8153-7348

Tang-Jie Nie https://orcid.org/0000-0003-2405-8904

Heng Liu https://orcid.org/0009-0008-4900-4025

Xue-Fei Ding https://orcid.org/0009-0008-2034-5459

Ying Huang https://orcid.org/0009-0004-7731-6916

Chun-Ce Guo https://orcid.org/0000-0003-3376-1116

Wen-Gen Zhang https://orcid.org/0000-0003-0946-8614

### Data availability

All of the data that support the findings of this study are available in the main text.
